# Molecular characterization of *Fasciola hepatica* and phylogenetic analysis based on mitochondrial (nicotiamide adenine dinucleotide dehydrogenase subunit I and cytochrome oxidase subunit I) genes from the North-East of Iran

**DOI:** 10.14202/vetworld.2016.1034-1038

**Published:** 2016-09-29

**Authors:** Saber Reaghi, Ali Haghighi, Majid Fasihi Harandi, Adel Spotin, Kourosh Arzamani, Soheila Rouhani

**Affiliations:** 1 Department of Parasitology and Mycology, School of Medicine, Shahid Beheshti University of Medical Sciences, Tehran, Iran; 2 Research Center for Hydatid disease in Iran, School of Medicine, Kerman University of Medical Sciences, Kerman, Iran; 3 Department of Parasitology, Medical Faculty, Tabriz University of Medical Sciences, Tabriz, Iran; 4 Vector-borne Diseases Research Center, North Khorasan University of Medical Sciences, Bojnurd, Iran

**Keywords:** Cytochrome oxidase subunit I, *Fasciola hepatica*, Iran, molecular characterization, nicotiamide adenine dinucleotide dehydrogenase subunit I, phylogenic

## Abstract

**Aim:**

Fascioliasis is one of the most zoonotic diseases with global extension. As the epidemiological distribution of *Fasciola* may lead to various genetic patterns of the parasite, the aim of this study is to identify *Fasciola hepatica* based on spermatogenesis, and phylogenetic analysis using mitochondrial (nicotiamide adenine dinucleotide dehydrogenase subunit I [ND1] and cytochrome oxidase subunit I) gene marker.

**Materials and Methods:**

In this study, 90 *F. hepatica* collected from 30 cattle at slaughterhouse located in three different geographical locations in the North-East of Iran were evaluated based on spermatogenetic ability and internal transcribed spacer 1 gene restriction fragment length polymorphism pattern. Genetic diversity and phylogenetic relationship using mtDNA gene marker for the isolates from the North-East of Iran, and other countries were then analyzed.

**Results:**

Partial sequences of mtDNA showed eight haplotypes in both genes. The phylogenic analysis using neighbor joining as well as maximum likelihood methods showed similar topologies of trees. Pairwise fixation index between different *F. hepatica* populations calculated from the nucleotide data set of ND1 gene are statistically significant and show the genetic difference.

**Conclusion:**

*F. hepatica* found in this region of Iran has different genetic structures through the other *Fasciola* populations in the world.

## Introduction

Fasciolosis is a zoonotic parasitic disease that occurring in domestic grazing animals, affected on livestock economy by decrease in output products. The financial burden is projected to be around two million US dollars annually [1]. The causative species most commonly implicated of fasciolosis are *Fasciola hepatica* and *Fasciola gigantica* that *F. hepatica* is distributed worldwide, although *F. gigantica* is fixed to warm parts and has been present in Africa along with South and Southeast Asia [2,3]. The two species have now been notable on the morphological criteria such as body size and shape; nevertheless, these requirements are not generally trusted due to the morphological variety within the species [4].

On the other hand, these species are meiotically diploid and produce sperm and store in the seminal vesicle (spermic), but some Asian *Fasciola* forms are meiotically dysfunctional and cannot produce sperm (aspermic) [5,6].

Two species may be identified using molecular methods by nucleotide sequences of nuclear ribosomal central internal transcribed spacer 1 (ITS1) and ITS2 [7-9]. In addition, DNA sequences of mitochondrial nicotiamide adenine dinucleotide dehydrogenase subunit I (ND1) and cytochrome oxidase subunit I (CO1) genes have been applied to analyze intraspecific phylogenic relations of *Fasciola* spp. [9,10].

It’s been thought that *F. hepatica* started in Europe and circulation of flukes depended on migration of livestock with individual colonizers [10].

Iran is a thorough country with various geographical ecology and existence of both species from cattle observed. There are several studies from Iran ruminant fascioliasis, especially in cattle and buffaloes centered on geography and weather variability [11]. Although some molecular studies have been conducted in several areas for genotyping of *Fasciola* species without a study on spermatogenetic ability in Iran [11], there is no useful finding based on populace design and genetic modifications of *F. hepatica* Iran.

The objective of this study was not only to identify of *F. hepatica* based on spermatogenesis and ITS1 marker by PCR-restriction fragment length polymorphism (RFLP), but also analyze their phylogenetic relationship with population from different parts of the world in the North East of Iran using ND1 and CO1 as mitochondrial makers with available GenBank records. Furthermore, genetic variability of *F. hepatica* foci of this region of Iran inferred by mitochondrial DNA sequences to get the parasitic gene flow among different populations was evaluated.

## Materials and Methods

### Ethical approval

This research was approved by the Faculty of Medicine, Shahid Beheshti University of Medical Sciences, Tehran, Iran.

### *F. hepatica* collections and spermatogenetic ability

**Figure 1:**
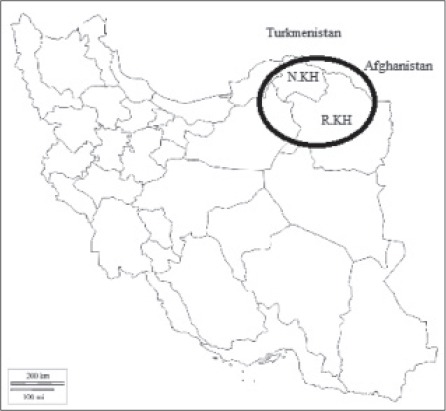
The provinces of the North and Razavi Khorasan in Iran.

A total of 90 adults of *Fasciola* specimens were collected from the bile ducts 30 cattle at slaughterhouse situated in three different geographical locations in the North East of Iran from January to September 2015. These cattle (*Bus taurus*) traditionally were nurtured. There are two provinces in the North East of Iran (North and Razavi Khorasan) bordering Turkmenistan and Afghanistan country (Figure-1). *Fasciola* flukes were washed in 0.9% saline solution and fixed in 70% ethanol between two glass slides and transported to the laboratory for further studies. The seminal vesicles of fixed specimens in the anterior part of body were removed and stained with hematoxylin–carmine solution, and then observed under a visual microscope to examine for the existence of sperm [12,13]. The posterior parts, excluding the uterus, which might contain sperm from other individuals, were used for total DNA extraction.

### DNA extraction and amplification

Total DNA was extracted from each fluke with a High Pure PCR Template Preparation Kit (Dynabio^®^, Takapouzist, Iran), according to the manufacturer’s instructions and stored at −20°C until use. DNA fragments of each target region were amplified by polymerase chain reaction (PCR) using a pair primer shown in Table-1 [14]. The total volume of reaction was 15 μl containing 1.5 μl DNA template, 5 μl distilled water, 10 pmol of each primers, and 7.5 μl master mix (amplicon). Reaction cycles consisted of an initial denaturing step at 94°C for 90 s, followed by 35 cycles at 94°C for 90 s, 53°C (ITS1) or 55°C (ND1 and CO1) for 90 s, and 72°C for 120 s, with a final extension at 72°C for 10 min using a gradient thermocycler. DNA fragments were analyzed by 1.5% agarose gel electrophoresis [9].

**Table-1:** The name and sequences of the primers used in this research.

Gene	Name	Sequence
ITS1	ITS1-F	5′-TTGCGCTGATTACGTCCCTG-3′
	ITS1-R	5′-TTGGCTGCGCTCTTCATCGAC-3′
ND1	Ita 10	5′-AAGGATGTTGCTTTGTCGTGG-3′
	Ita 2	5′-GGAGTAC GGTTACATTCACA-3′
CO1	Ita 8	5′-ACGTTGGATCATAAGCGTGT-3′
	Ita 9	5′-CCTCATCCAACATAACCTCT-3′

ITS1=Internal transcribed spacer 1, ND1=Nicotiamide adenine dinucleotide dehydrogenase subunit I, CO1=Cytochrome oxidase subunit I

### RFLP of amplified DNA (PCR-RFLP)

The ampilcons of the ITS1 region were examined by the PCR-RFLLP method. Briefly, the reaction level of 10 μL contained 5 μL of PCR products with approximately 680-bp fragments, 1 U of the *RsaI* restriction enzyme, and 1 μL of manufacturer supplied reaction buffer (Cinagen^®^, Iran). After incubation at 37°C for 3 h and heat inactivation of *RsaI* at 65°C for 15 min, the digested DNA samples were analyzed by gel electrophoresis [8].

### Sequences and phylogenetic analysis

Products of ND1 and CO1 of isolates sequenced by Bioneer Company utilizing the same primers, which were found in the PCR. The sequences were aligned and in contrast to those of existing sequences from the region, linked to *Fasciola* spp., available in the GenBank, utilizing the Chromas 2.2 and multiple alignments were performed with data linked to *F. hepatica* from Iran and other countries deposited in GenBank. Phylogenic analyses predicated on NDI and COI sequence data were conducted by maximum likelihood using MEGA6 [15]. All characters were run unordered and equally weighted. Alignment gaps were treated as missing data. Bootstrap analyses were conducted using 1000 replicates.

### Genetic diversity indices

Diversity indices (Haplotype diversity; Hd and nucleotide diversity: π) and neutrality indices (Tajima’s D and Fu’s Fs tests) were estimated by DnaSP software version 5.10 [16]. The people genetic structure was analyzed by Arlequin version 3.11 [17]. The degree of gene flow (gene migration) on the list of populations was evaluated utilizing a pairwise fixation index (Fst) [18].

## Results

**Figure 2:**
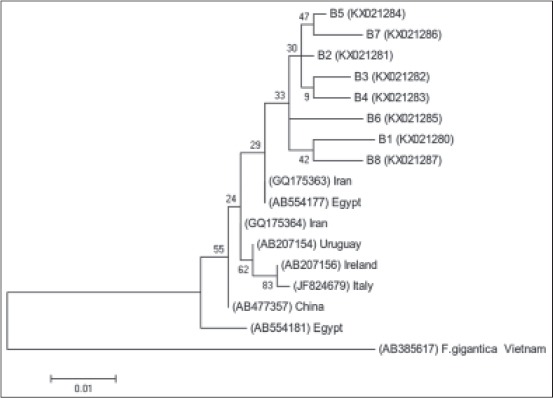
Phylogenetic relationship based on nicotiamide adenine dinucleotide dehydrogenase subunit I sequences of Fasciola hepatica from the North East of Iran. The tree constructed by MEGA6 using neighbor-joining analysis. Scale bars indicated nucleotide substitutions per site. Fasciola gigantica was used as outgroup.

**Figure 3:**
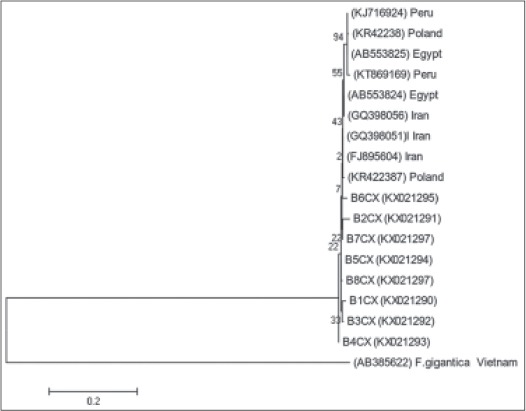
Phylogenetic relationship based on cytochrome oxidase subunit I sequences of Fasciola hepatica from the North East of Iran. The tree constructed by MEGA6 using neighbor-joining analysis. Scale bars indicated nucleotide substitutions per site. Fasciola gigantica was used as outgroup.

All 90 sample specimens had many normal sperms in the seminal vesicles and were spermic species. Based on the RFLP fragment pattern in ITS1 region, they exhibited the *F. hepatica* type. ND1 fragments (approximately 535 bp) and CO1 fragments (approximately 438 bp) were amplified for several specimens. Partial sequences of ND1 and CO1 showed 26 and 11 variable sites, respectively, and also yielded eight haplotypes in both genes and high diversity indices in ND1 gene (Table-2). The nucleotide sequences for every haplotype were deposited in GenBank under following accession numbers: KX021280-KX021299. The profiles of *F. hepatica* haplotypes based on ND1 and CO1 genes and type of nucleotides in the North East of Iran shown in Table-3. Phylogenic analyses based on ND1 and CO1 sequence data were conducted by neighbor-joining (NJ) using MEGA6 with *F. gigantica* designated as an outgroup showed in Figures-2 and 3. Pairwise fixation index (*Fst* values) between different *F. hepatica* populations calculated from the nucleotide data group of ND1 gene are statistically significant and show the genetic difference in pairwise population (Table-4).

**Table-2:** Haplotype diversity and nucleotide diversity of *Fasciola* fluke in North East of Iran based on ND1 gene.

Species	Population	Diversity indices				Neutrality indices	
	
		n	Hn	Hd±SD	π	Tajima’s D	Fu’s Fs statistic
*F. hepatica*	North East of Iran	90	14	0.997±0.003	0.01552	−1.3724*	−2.59

* Statistical significance: Not significant, p>0.1. Hn=Number of haplotypes, Hd=Haplotype diversity, Nd=Nucleotide diversity, SD=Standard deviation, *F. hepatica=Fasciola hepatica*, ND1=Nicotiamide adenine dinucleotide dehydrogenase subunit I

**Table-3:** Profiles of *F. hepatica* haplotypes and their accession no in the North East of Iran.

Location (Province)	Bojnurd and Shirvan (North Khorasn)	Maneh and Samalghan (North Khorasn)	Ghochan (Razavi Khorasn)
Host (number)	12	8	10
Number of flukes	38	25	27
Sperm in seminal vesicles	+	+	+
ITS1-RFLP	*F. hepatica*	*F. hepatica*	*F. hepatica*
Haplotype (accession no.)			
ND1	B1ND (KX021280) B2ND (KX021281) B3ND (KX021282) B4ND (KX021283) B5ND (KX021284)	B3ND (KX021282) B6ND (KX021285)	B6ND (KX021285) B7ND (KX021286) B8ND (KX021287)
CO1	B1CX (KX021290) B2CX (KX021291) B3CX (KX021292) B4CX (KX021293)	B5CX (KX021294)	B6CX (KX021295) B7CX (KX021296) B8CX (KX021297)

ITS1=Internal transcribed spacer 1, ND1=Nicotiamide adenine dinucleotide dehydrogenase subunit I, CO1=Cytochrome oxidase subunit I, RFLP=Restriction fragment length polymorphism, *F. hepatica=Fasciola hepatica*

**Table-4:** Pairwise fixation index (*Fst* values) between different *F. hepatica* populations calculated from the nucleotide data set of ND1 gene.

Population	Population				

	Iran (this study)	Egypt	Peru	Asia*	Europe**
Iran (this study)	-				
Egypt	0.98329	-			
Peru	0.98516	0.98892	-		
Asia*	0.89128	0.89282	0.01768	-	
Europe**	0.78107	0.78042	0.00250	−0.06162	-

* Asia: China, Thailand, Japan,

** Europe: Italy, Poland. All values are statistically non-significant (p>0.05). ND1=Nicotiamide adenine dinucleotide dehydrogenase subunit I, *F. hepatica=Fasciola hepatica*

## Discussion

Microscopical observation after staining revealed that spermic *Fasciola* occurred in the North East of Iran. There are always a few studies in Iran that concentrate on spermatogenesis in Fasciola *sp*. Ashrafi *et al*. reported *F. hepatica*, *F. gigantica* and intermediate forms in the endemic region of the North East of Iran (Gilan province) based on morphological and phenotypic analysis of *Fasciola* flukes [4]. In this study, all of the samples were spermic *F. hepatica* with spermatogenetic ability.

Epidemiological patterns are crucial and the important factors in differentiation of *Fasciola* species. Despite the fact that phenotypic criteria have been considered to be one of the useful criteria for discrimination of species in Fasciola [19], but molecular approaches clarify of global genetic diversity and distinguish intraspecific relations. RFLP methods using ITS regions were used to identify the *Fasciola* species and has been extensively validated [20,21]. This method has been used in a number of studies in Iran [22,23], but studies showed that molecular phylogeny with mtDNA, including ND1 and CO1, could be effectively useful for proper differentiation of haplotypes [9,14].

The *Fst* values showed that *F. hepatica* population in three continents was genetically different from one another centered on NDI region (Table-3). These results could be related to the current presence of different haplotypes of investigated populations. Furthermore, this implies that here’s no transfer of alleles in one population to another population through immigration of *F. hepatica*.

The phylogenic analysis using NJ in addition to ML methods showed similar topologies of trees. Some reports from Iran were detected *Fasciola* *sp*., and constructed phylogenic trees using nuclear rDNA and declare that because of variation in this region; it’s inadequate to separate your lives of *Fasciola* species for resolving the taxonomic problem [24-26].

ND1 haplotypes of this study show a high range of diversity, but they belonged to one clade in shown phylogenetic tree. The phylogenetic trees showed relationship of isolated of other regions in Iran, but more studies and the population analysis need to get exact classification pattern.

## Conclusion

This study demonstrated that *F. hepatica* found in this region of Iran has different genetic structures through the other *Fasciola* populations in accordance with pairwise fixation index, but to complete and find genetic diversity other molecular studies from another region of Iran is necessary.

## Authors’ Contributions

SaberR and SR together have designed, planned and conducted this research. AH, MFH, and AS assisted in the execution. KA collected specimens. All authors have read and approved the final version of the manuscript.
